# Food Education, Cookery Books and School Canteens in the Fight against Malnutrition: The Case of the Spanish Edalnu Programme (1961–1986)

**DOI:** 10.3390/ijerph19095427

**Published:** 2022-04-29

**Authors:** Maria Tormo-Santamaria, Josep Bernabeu-Mestre

**Affiliations:** Carmencita Chair of Gastronomic Flavour Studies, Balmis Research Group in History of Science, Health Care and Food, University of Alicante, Sant Vicent del Raspeig, 03660 Alicante, Spain; josep.bernabeu@ua.es

**Keywords:** malnutrition, food education, school canteens, cookery books, Programme Edalnu, Spain, second half of the 20th century

## Abstract

The Spanish population completed its nutritional transition in the 1960s and 1970s, when it overcame the problems of malnutrition. Among the initiatives that made this possible, the Food and Nutrition Education Programme (Edalnu) (1961–1986) stands out. In addition to correcting the negative influence exerted by ignorance to nourish oneself correctly, it was intended to prevent the problems of overfeeding that most developed countries showed. The objective of this research addresses, in this context and from the parameters of applied history, the condition of the complementary pedagogical instrument that the Edalnu awarded to the school canteen in the fight against malnutrition, as well as the nutritional, dietetic, culinary and gastronomic criteria used for its operation. The results show that the school canteens sought to reinforce the food knowledge acquired in the classroom. Based on the dialogue between chefs and experts in nutrition and dietetics, balanced meals adapted to regional gastronomic diversity were prepared, which helped to promote, in line with current criteria, healthy and sustainable eating habits through traditional plant-based recipes, with a predominance of seasonal and local products, and with a complementary contribution of ingredients of animal origin.

## 1. Introduction

Throughout the 1960s and 1970s, the Spanish population completed its food and nutritional transition with the resolution of malnutrition problems and the achievement of the recommended nutritional intake [1,2]. After the autarchic period and the Civil War, a process of economic development began in the 1960s which was not exempt from inequalities, also present in the field of food and diet [3,4,5]. As indicated in a review of the surveys on diet and the nutritional status carried out in Spain in the 1940s, 1950s and 1960s; in the 1970s, there was still clear socio-economic inequality in terms of the consumption of animal proteins and vitamins, with the agricultural labourers and industrial workers being the worst fed [6].

The situations across the Spanish territory were clearly diverse, but the problem was not so much due to a lack of resources, which was experienced by certain groups and aggravated by the deficiencies of the Spanish agri-food sector [3,5], but to the challenge of educating the population in which foods they could afford and which were the best for an adequate diet. Initiatives aimed at addressing this challenge were implemented within the framework of some of the conventions and international agreements signed during the Franco dictatorship. In 1954, the School Food and Nutrition Service (SEAN) was created. This organisation was responsible for distributing the so-called American Social Aid, more specifically, milk, among Spanish school children. In 1961, the activities of the Food and Nutrition Education Programme (Edalnu) began, thanks to the technical support of the FAO and the economic support of UNICEF. The Edalnu was the largest initiative in food and nutrition education developed in Spain in the 20th century [4,7,8,9,10,11].

As indicated by the director of the Edalnu, Doctor Palacios, its implementation responded to the need to overcome the negative influence exercised by an “ignorance” of a deficient diet exhibited by wide sectors of the Spanish population. It also sought to prevent the overeating problems which had been experienced and continued to prevail in the “developed” countries, by promoting better eating habits [12]. As we can deduce from his testimony, the Spanish authorities were aware of the need to attempt to prevent the problems of excess weight and obesity which had begun to emerge in those Western countries that had fully completed their food and nutritional transitions [13,14].

Having overcome the problems of malnutrition by default, at the end of the 1970s, the majority of the Spanish population was able to satisfactorily cover their nutritional requirements with a caloric profile that almost perfectly matched the recommendation of the international organisations [15]. However, this situation, which was ideal from an epidemiological and nutritional point of view, was not maintained. Some years later, in the 1980s, the nutritional panorama of Spain was marked by the emergence of problems associated with overeating [16,17]. At the same time as the problems of malnutrition characteristic of the pre-transitional stage were being resolved, as previously commented, other post-transitional problems arose related to high-calorie diets, a greater consumption of animal fats and free sugars or the lower intake of high-fibre foods. All of these factors explain a considerable part of the changes that took place alongside Spain’s epidemiological transition [18,19].

Similarly to the international context [20], in the case of Spain, over the last few decades, excess weight and obesity have become a major public health problem and one which particularly affects the child population for its consequences in adulthood. In 1985, the percentage of overweight Spanish children was 15%. In 2019, according to the results of the Aladino study, this percentage was almost 40%. More specifically, 41.3% in the case of boys and 39.7% in the case of girls [21].

The epidemiological and nutritional transition experienced by the Spanish population at the end of the 20th century and beginning of the 21st century has been characterised by an absence of effective preventive policies, particularly in the field of food and nutritional education [9,18]. Despite its success and the scope and dimension that it attained [7,8,9,10,11,22], the Edalnu began to scale back its activities at the end of the 1970s and early 1980s, at a time when a programme of this kind was needed more than ever, as the number of cases of excess weight and obesity began to rise. Among the factors that explain the dynamics of the deceleration of the Programme, we can refer to both the change in the ministry responsible for it, from education to health, and to the impact of transferring the responsibility of promoting health to the autonomous regions after the constitutional reform of 1978 and the development of the State of the Autonomous Regions [9,10,23]. Although the Strategy for Nutrition, Physical Activity and the Prevention of Obesity (NAOS) was implemented in 2005 with the objective of “improving dietary habits and promoting the regular practice of physical activity of all citizens, with particular emphasis on prevention during childhood”, the figures of excess weight and obesity have continued to rise [24]. Moreover, the lack of an adequate coordination between the different authorities has been apparent in, for example, school nutritional policies [25,26].

Among the strategies proposed to mitigate the problem of excess weight and obesity and, more specifically, that affecting children, is the role of school canteens and their capacity to feed, nourish and educate within a framework of a traditional (local cuisine and gastronomy), tasty, participative and healthy diet [27,28,29,30,31]. This was the case, of some of the strategies designed by the Edalnu programme, with respect to canteens, recipe books and school meals [7].

The objective of this study is to address, from an applied history perspective and in the framework of the fight against malnutrition, the educational activities carried out by the Edalnu in school canteens and the culinary and gastronomic offer that they provided. It seeks to analyse how the canteens were integrated into the rest of the teaching activities in food and nutrition education which the schools had to develop; the criteria used in preparing the recipe books which provided the base for creating the dishes or meals; the selection of ingredients and condiments and their presentation, and their suitability to ensure an adequate diet and nutrition. The experience of programmes such as Edalnu can provide elements on which to reflect and interesting proposals for current school meal policies aimed at preventing excess weight and obesity, through the promotion of healthy and sustainable dietary, culinary and gastronomic habits [26].

## 2. Materials and Methods

The main primary sources used are, on the one hand, documents (monographs and leaflets) related to the school canteens, with recipe books and school meals and menus published by the Edalnu [32,33,34,35,36]. On the other hand, we have also consulted documents from some of the organisations which collaborated in its activities, such as the School Food and Nutrition Service and the General Directorate of Primary Education or the National Institute of Hygiene, Food and Nutrition and the Nutrition Section of the Clinical Research Institute [37,38,39]. In addition, other material generated by the Edalnu related to other training activities on food and nutrition education has also been consulted [12,40,41,42,43,44,45,46,47,48,49,50,51].

Most of these are documents published by official bodies that may have been subject, in the first stage covered by the study, to the ideological control exercised by the Franco dictatorship [8]. To this limitation, the fact must be added that data were not always provided that allowed us to contrast the extent to which the proposals collected in the analyzed sources were put into practice.

As well as revealing aspects related to the model and objectives of the school canteens, the analysis of the sources also allows information to be gathered regarding the criteria that guided the preparation of recipe books and meals (including the ingredients and the explicit mention of local or proximity products). In addition, it enables the collection of data regarding culinary techniques, gastronomic and nutritional recommendations, culinary traditions and regional cuisines, among other aspects.

Conducting the research over a period of time reveals the change in priorities of the Edalnu which is reflected both in the recipe books and in the objectives established for the school canteens. The focus shifted from resolving the problems of malnutrition by default in the early documents published in the 1960s and 1970s to addressing the emerging problems of excess weight and obesity in the 1980s [15].

## 3. Results and Discussion

In accordance with what was occurring on an international level [52,53], the teaching system developed by Edalnu for schools in terms of educational activities in food and nutrition was based on the development of three basic principles [38,39]: the acquisition of knowledge (the informative stage), the creation of habits (the formative stage) and the development and communication of attitudes (the attitudinal stage). The programme consisted in the preparation of six educational units related to teaching about food and nutrition, the promotion of a dietary supplement, namely milk, and the promotion of canteens, vegetable gardens, farms and school clubs. The teaching framework was to be implemented by the teachers, but with the active participation of the students and the cooperation of the parents and families. In order to implement the programme, a teaching support infrastructure was set up with the creation of a Primary School Council made up of fifty-six National Schools of Food and Nutrition Education: three in Madrid, two in Barcelona, Oviedo, León and Valencia and one in each of the remaining provinces [12]. These schools were run by national teachers with an Edalnu Diploma after receiving the corresponding training within the framework of the instructor network that designed the programme [10].

Within this context and in line with what is currently being demanded [54], the school canteen acquired the condition of a teaching instrument and educational complement, as it reinforced the knowledge acquired in the classroom on food and home economics [39]. Through a broad series of teaching proposals on food and nutrition, the composition of the different foods was explained with their classification, the immediate principles, the vitamins and minerals and the seven food groups, using a dietary guide known as the food wheel [46,49,55,56]. Moreover, in order to consider other extra-nutritional elements that could influence dietary habits or preferences, importance was also given to the wealth of local and traditional recipes, to analysing the nutritional value of typical Spanish dishes and to how they were prepared [32,33,35,51].

The home economics lessons were organised into five groups: (a) basic notions of nutrition in order to achieve a balanced diet; (b) knowledge on how to correctly select, purchase and store food products; (c) how to cook and prepare them, preserving their nutrients; (d) domestic preservation systems and (e) a complementary section on optimising economic resources and time management [47]. Home economics was not focused solely on the kitchen. With a gender bias [8], it was about training girls as future housewives, to educate them in good work habits, provide them with order and selection criteria and help them solve their problems with the resources available to them [45,48].

A few months before the Edalnu programme was implemented, in 1960, the Ministry of National Education had initiated a school canteen plan, which had the primary objective of dispelling the old concept of the school canteen as a place that served the most needy or where children could “get fed” [37,39]. The plan contemplated a dietary proposal elaborated jointly by the School Food Service and the technical experts of the National Institute of Hygiene, Food and Nutrition [37]. Previously, and in order to determine the average dietary requirements of Spanish school children, two surveys were conducted in public schools in Madrid. The first was carried out in an average neighbourhood and the second in a poorer one. A third survey was conducted in a well-off neighbourhood of Madrid [37]. Based on the data obtained referring to the type of food that the surveyed children received, a series of meals were created, adapted to the ages of the children (see Table 1) which covered their nutritional requirements and complemented the family diets with recipes based on the products available, including those provided by the American Social Aid, known as ASA food. These meals were tested in a “pilot” canteen, installed in the “Patriarca Eijo Garay” school and the summer camps for school children run by the Ministry of Education [37]. 

The eighteen meals proposed were characteristic, particularly in the case of the first courses, of the culinary language of traditional Mediterranean food culture [57]. The principal components were cereals, pulses and vegetables, while meat products were given a more complementary role. These meals reflected the food, culinary and gastronomic context of the Spanish population in the light of the changes occurring in the 1970s and 1980s and which led to a Westernisation of the diet [58].

The meals were accompanied by a calendar and were grouped by term [37]. They were complemented with recommendations about condiments and the presentation of the recipes and highlighted the protective properties of fruit or the powdered milk provided by the American Social Aid. It was very important “to prepare food with care, making it tasty and appealing,” and to present it carefully “because changing the taste and appearance of a delicacy can break the monotony and benefit the well-being and delight the school children.” Adding condiments to improve these aspects was permitted. However, in no case “could the basic recommended foods be omitted or substituted.” Neither could “chemical procedures that reduced or neutralised the nutritional value of the foods or which could endanger the health of the children” be used [37]. It should be remembered that in this respect, the issue of additives and the quality control of the foods was the object of debate within the framework of the developmental policies that Franco’s dictatorship was beginning to promote. In the early 1960s, the first steps were taken to establish a Food Code [5].

As previously indicated, the importance of fruit was highlighted when the meals were designed. The consumption of oranges was recommended [44] “to ensure that the children’s intake of Vitamin C was higher than the essential amount” during the winter. During the months when there were no oranges, the consumption of melon, watermelon, bananas, apricots, grapes, apples, cherries and peaches was recommended, ordered in accordance with their nutritional value. In any case, the need to consider the production of each district was highlighted so as to promote the consumption of proximity products [36] as is the case in the current methodology in learning [59,60].

The school canteen plan of 1960 also contemplated assessment actions of results. It recommended the periodical establishment of “detailed information revealing the liking and tastes of the school children with respect to each of the meals.” Furthermore, the results obtained were gathered in the form of anthropometric indicators (weight and height) which were recorded in the files of each diner (boy or girl who ate in the canteen) [37] Although we have not been able to find documents referring to the collection and analysis of these data, specifically the anthropometric indicators, other publications promoted by the Edalnu or the SEAN indicate an improvement in the height of school children, coinciding with the implementation of school canteens and the distribution of the food supplements (powdered milk from the American Social Aid and later, bottles of milk) in schools [4,40].

The evolution and development of the school canteen plan was analysed in a publication in 1968 [33]. In the school year 1965–1966, a total of 5740 canteens were operating in state and non-state primary schools, supervised and directed by the SEAN and integrated in the National Network of School Canteens, which provided a service to more than 200,000 primary school children. Furthermore, in each province, there was a “pilot” canteen that constituted a model and stimulus for the rest [33]. The same document insisted on the need and opportunity to conduct a periodical assessment of the results. The aim was to find out the acceptance of the meals by the children who came to the school canteens and to monitor the evolution of their height and weight. Moreover, in line with the objectives of Edalnu in terms of the school canteens [36], their purpose and goals were indicated [33].

The school, through the school canteens, had to compensate for the impact that the incorporation of women into the world of work had had on the formative capacity of families in terms of food. Together with the improvement in their nutritional status, “often not due to a meagre diet but to a defective one,” the school canteen should “educate the children about what they should eat, how they should eat and why they should eat.” School canteens were to serve to increase school performance and the capacity to work as a result of the improved nutritional status. They also became a practical lesson on a correct and balanced diet; to contribute to the promotion of civic and social skills through the conviviality of eating in company; to acquire hygienic habits related to the act of eating, and in the case of the female students, as already indicated, with a clear gender bias, the school canteen become a practical class of home economics, because by collaborating with the tasks in the dining room, they could “acquire skills that will be very useful in their future as housewives” [33].

The school canteen had to serve for the practical application of theoretical knowledge about food acquired in the classroom [33]. To develop “globalised teaching units”, enabling the school children to address in a realistic way.“ From issues included in their academic tasks: notions of human physiology and natural science, to physics and chemistry phenomena, the geography of food production, vocabulary and language exercises, buying and administration aspects or hygiene and sanitary regulations.” Or, as indicated in another of the sources consulted [32], “to show them the reality of the food and nutrition theory that they are taught, explaining to them why they are given each food, in a highly useful practical exercise which has a greater impact than a mere theoretical presentation.” It was a strategy of learning by doing, which was also a feature of the educational activities such as those conducted, in collaboration with the Edalnu, by the Agricultural Extension Services in the school vegetable gardens [11,42] and which are highly similar to the current methodology in learning environments [61].

As a complementary teaching resource, the school canteen had a felt board, allowing the meals to be updated daily, indicating the composition of the food [33]. It had two parts. On one side was a guide to the food wheel with the seven groups and drawings of the most representative. In this way, each day, the food that would be eaten was separated and the children could see the groups to which the foods belonged. The other side of the felt board displayed drawings of the food and the prepared dishes. One of the objectives sought by the school canteens was to create the habit among the children of eating a varied and balanced diet which included foods from the seven groups and contributed the necessary nutritional substances in optimum amounts [39].

With respect to assessing the degree of satisfaction of the school children with the proposed meals, in 1966 [32], the reports of the Edalnu diploma holders who coordinated the school canteens highlighted the problem of their uniformity and the consequences of not contemplating the diversity of the culinary and gastronomic traditions of the different Spanish regions [32]:

“The experience of several years has shown that the meals have some defects, the most important, perhaps, being their uniformity across the whole of Spain. Some degree of uniformity is undoubtedly necessary to avoid excessive deviations, which could lead to considerable errors. However, at the same time, the variety of the Spanish regions is such that it is difficult to subject them to a single model. The differences in climate, customs, and idiosyncrasies means that meals that are well-received in some places are not to the liking of others. What is appealing for someone from Valencia would not arouse much enthusiasm in a Basque child. And the meal which included gazpacho has been almost unanimously criticised in the part of the country north of Madrid and praised in the part south of the capital.”

It was not a question of regionalising the meals, as, according to several of the documents referring to them [34,35,36], one of the objectives of the school canteens was to “teach the children to eat foods that they are not accustomed to, drawing the population out of the dietary routine in which they were immersed” [32]; however, it was advisable to include regional dishes, “which would undoubtedly please the children of that region and which, in the other regions, enabled the celebration, from time to time, of Valencia day with a paella or a Canary Islands day with rancho canario (Canarian soup), etc.”. Furthermore, as also indicated in the leaflet [31], the regional dishes “could be cheaper in each area and sometimes easier to prepare than the general dishes.” They also favoured the consumption of local products, which is also a feature of today’s food guides to promote healthy, safe, tasty, sustainable and socially accepted foods [62,63].

In fact, together with the updating of the twenty classic dishes, corrected in accordance with the observations collected “through many meetings with provincial delegates, Edalnu diploma holders, canteen directors, etc.”, in the 1966 leaflet, twenty-eight regional dishes were included “proposed by holders of the diploma in Food and Nutrition Education of different areas” and which were accepted with very few modifications (see Table 2). According to the authors of the text, there were “enough so as to offer a choice to those canteen directors who, for whatever reason, wished to deviate from the twenty basic meals or dishes from time to time” [32].

The demand for regional dishes was also driven by one of the organisations which collaborated with the Edalnu, the Women’s Section of the Falange, the female branch of the only political party of a fascist ideology, which supported the Franco dictatorship. Although they were accompanied by the political and gender discourse that characterised the organisation [8], the recipe books published, in addition to offering useful tips for beginners, covered some basic foods. The grouping of the products by season helped to order the recipes and provide seasonal dishes. The idea was to offer quality dishes that could be made with economic and accessible ingredients and which were easy to prepare [57].

As in the case of the meals of 1961 [37], as well as showing the diversity of the culinary traditions across Spain, the regional dishes included in the text of 1966 [32] followed the pattern of two courses complemented with fruit and milk or other dairy products. There was a predominance of spoon dishes and their cooking technique. Cereals, pulses and vegetables continued to provide a vegetable base to the lunches with a complementary presence of meat and fish, mostly in the second courses. On the whole. they had the basic characteristics of the traditional Mediterranean diet [57].

The proposals of meals elaborated in the 1960s were followed by others created in the 1970s, with the primary objective of putting greater emphasis on condiments and the presentation of the food [34].

“In response to the overall growing interest to consider condiments and the presentation of food, we have seen the need to create a recipe book that partly helps to resolve this problem […] if condiments are important, the presentation of food is even more so […] things as simple as potatoes, carrots and sardines can appear very different in a well-presented dish, which are in no way inferior to the best delicacies […] we can find a hundred different kinds of recipes which to alter the daily routine of customary and outdated stews […] are made economically, considering their nutritional value and the perfect harmony of the ingredients […] each recipe can sufficiently cover the daily needs of a normal person with respect to their nutritional intake […] we hope that the readers find formulas that they did not know before and new ways of preparing their dishes.”

As previously mentioned, the condiments and cooking of the dishes constituted one of the major concerns, as “the most perfect dish will be systematically rejected by the children if whoever has to prepare it does not know how to condiment it in a pleasing way or does not bother to do so.” Therefore those responsible for the dietary and nutritional aspect of the canteens identified the need to establish close collaboration with the cooks [39]:

“The experience that we have gained fully confirms this. The same dish can be loved in one canteen and rejected in another, simply because the former has a competent cook and the latter does not. Hence, people who know how to cook must work with the bases provided by the doctors (who know little about cooking) in order to put our suggestions into practice. On the other hand, the cooks can indicate modifications: replace one vegetable with another, add a certain garnish, remove a sauce, based on practical reasons, such as the work involved in preparing a dish, its best presentation, fuel consumption, etc.”

As we can see, similarly to the approaches of the present day [64], the way in which the recommended meals were put into practice with the creation of recipes was through dialogue between dietary and nutrition experts and cooks (experts in the culinary arts). The guidelines also advised that the meals were prepared according to the culinary and gastronomic parameters of the environment of the schools [32]:

“Therefore, this collection of meals, compiled by a doctor, has to be implemented in practice in the form of recipes, to which, maintaining the foods and the amounts indicated, condiments are added (with little nutritional value but which liven up the dish). Furthermore, the cooks must give advice about the ways (there are, naturally, more than one) that each of the dishes can be prepared, always seeking to ensure that they are similar to those used in the homes of the children. In other words, simple, home-style cooking which children easily accept and which makes it easy for mothers to prepare dishes that the child has been given at school.”

In the 1980s, the meals proposed for school canteens were simplified [36] and greater focus was placed on the problem caused by obesity in the school environment [35] and, again, the nutritional value of typical Spanish dishes was emphasised [51].

Similarly to what had been indicated in the cookery books produced at the end of the 1960s and in the 1970s, it was stressed that the food served in the school canteens should also take into account the regional habits. This did not mean that the children of the east coast would eat rice every day of the month prepared in its many different forms, or that the Andalusian or Extremaduran children would not eat anything other than gazpachos and stew. However, at least fifty per cent of the days that they ate in the canteen, they should be served regional dishes and, if possible, with the incorporation of different ways of culinary preparation and the inclusion of other less-known foods [36]. The objective was to use the variety of resources offered by the culinary and gastronomic traditions of the different Spanish regions [36]:

“Spain is a country with an enormous variety of products and a very rich gastronomy which allows for excellent diets based on tradition. Slight modifications of these diets make them perfect. We should ensure that school children know them and do not reach adulthood without ever having eaten an aubergine, a courgette or know the ingredients of a menestra (mixed vegetables) or the taste of a lamb or cod stew.”

The manual published in 1985 [35] included a total of 21 meals which were “easy to prepare and had a high nutritional value”, six of which were included in the category of “combined dishes”, given that they could be presented on one plate. The remaining fifteen were more traditional dishes (Table 3).

The informative sheets provided to educators stated that the meals served in the school canteens had to respond to the nutritional needs of children of school age, respect the gastronomic characteristics of each region (not meaning that the children would always eat the same thing or not eat other types of necessary foods), to be presented and garnished in a simple but attractive way, with a moderate use of salt, sugar and spicy sauces, and not abuse fried foods. Another objective was to enhance the sensory satisfaction of the children in order to stimulate their digestive juices and contribute to a better metabolisation of the food. Moreover, special attention had to be given to the environment in which the children ate (place, available space, noise, fumes and company at the table), the time allocated for eating, the presentation of the dishes, the temperature at which it should be served and the sequence in the serving of the food in order to avoid a long space of time between courses [36]. As we can see, this approach is highly in keeping with the requirements that the school canteens should meet in the present day [64].

All of these recommendations were complemented with a proposal for a conventional or traditional meal structure. A monthly assessment was proposed, taking into account the supply of food on the market (seasonality), without forgetting the versatility of frozen products. Based on the study of supply with the corresponding prices and ease of preparation and storage, a monthly meal plan was formulated which was flexible but safeguarded their nutritional value [36].

The recipes were interspersed with pieces of advice in order to ensure the quality and safety of their preparation [35]. It insisted on the benefits of combining pulses with cereals and vegetables in order to ensure the intake of proteins with a high nutritional value. However, most of all, it advised against the things that should not be done and those which should be prioritised [35]. A sweet dessert (cake, ice-cream, flan) should not substitute a piece of fresh fruit which, in turn, could not be replaced by a compote, fruit jam or baked fruits. Wherever possible, salads should be introduced as garnish. A balance should be sought in the meals in terms of nutritional value, “a meal composed of macaroni, Spanish omelette and a cake would be incorrect as it contains too many carbohydrates”. Milk, dairy-based desserts and cheese should be included in the meal as often as possible; orange should be prioritised of all the fruits as they “have a better ratio between price and vitamin content”. Finally, the parents of the children should be provided with information about the school meals with a full explanation so as to ensure their collaboration and support.

In addition to this type of advice, a series of suggestions were made for planning the meals, taking into account: (a) for whom they were to be made. In this sense, it was necessary to determine the nutritional needs, dietary habits and the structure of the full meal, local customs, etc., of the children; (b) the number of meals that had to be served in the school each day; (c) the ease with which the food was prepared and the culinary knowledge of those responsible for the school meals; (d) the money available for purchasing the food and (e) the type of service to be provided: self-service system with help of waitresses, with the collaboration of the children, etc. [35].

They comprised a series of considerations which suggested the existence of a diverse range of possibilities for schools in running the canteens. These circumstances could have been behind the proposal of combined dishes (easier to prepare and without the need of recurring to more sophisticated culinary techniques) which complemented the traditional dishes but had a lower nutritional value and were less healthy, as suggested in the document [35]. The introduction of the combined dishes represented a substantial change in the culinary criteria. The juxtaposition on the plate places the meat or fish as the main feature and the cereals, pulses and vegetables as complementary condiments. This breaks one of the healthiest elements of culinary traditions such as the Mediterranean diet: the intake of legume proteins as a complement to cereals, with a very moderate consumption of animal proteins [57]. Furthermore, we should not forget that the cooking technique facilitated the mixture of tastes, flavours and textures, making the meals more palatable and more attractive as well as more balanced and complete from a nutritional point of view, two of the elements that are fundamental in terms of children’s diets [57,64].

In fact, it was this meal proposal elaborated in 1985 [35] that explicitly mentioned the issue of obesity. As well as explaining how overeating could lead to an obesity problem in the child which would be difficult to overcome in adulthood, it also warned of the psychological consequences: “Obese children feel self-conscious among their classmates and are often the target of jokes and teasing which affects their personality, making them shy and withdrawn.”

With a very modern approach [65], it affirmed that the key to avoiding excess weight and obesity resided in providing a balanced diet based on the correct variety and amount of food and that natural food constituted the basis of health: “Minerals and vitamins should not be taken in pill or syrup form but through fruit, vegetables, salad and dairy products” [35].

The traditional dishes proposed for lunch (Table 3) were made up of a basic dish, a second course and a third course or dessert. In the first case, the basic ingredients were rice, pulses, potatoes and vegetables, to which meat or products derived thereof, fish, eggs etc., could be added always to complement or complete. In other words, the same traditional culinary language as the meals of 1961 and 1966 was maintained, as were the parameters of the Mediterranean diet in many of the dishes. The dominant value of the first course was the energy intake and its objective was to cover the quantitative needs, as didactically explained in the document [36]:

“[…] it is important that the children get used to eating it because the energy needs are the first that should be covered if we want the providers of protein (meat, eggs, fish) to fulfil their function in the body of forming new tissue and favouring growth. If this is not taken into account, the organism with use the proteins to resolve its energy needs and the diet will be unbalanced, anti-physiological and expensive.”

The basis of the second course was meat or offal or other meat products, fish or eggs. These foods were interchangeable and had to be eaten in moderate amounts because, as indicated in the source consulted, “hunger should not be satisfied with proteins.” It was recommended to garnish the dish with salads and vegetables or a good sauce, with the reminder that potatoes were usually preferred by school children, so they should not be the only garnish [36].

It proposed that the third course or dessert was fruit-based as, together with its vitamin (particularly if it was fresh) and fibre content, it had very few calories and a different consistency and texture to other foods, rendering it pleasant and refreshing. Furthermore, as the information sheets for educators explained: “It favours chewing and, to some extent, it clears our teeth of possible residues” [36].

Finally, as a possible complement, it indicated the possibility of complementing the three courses with a dairy product in order to reinforce the diet with calcium, B2 vitamins and proteins with a high biological value. In this respect, the document read, “calcium and phosphorous are essential in the growth phase and dairy products contain them in a large amount and in a chemical form in which they can be absorbed and used” [36].

The proposal of a lunch made up of three dishes in the school canteens responded to the fact that in Spain, lunch is the main meal of the day. Not taking this into account “would run the risk of children with a poor nutritional condition as they ate a very simple breakfast and a light evening meal” [35]. The initiative, therefore, sought to cover at least 40% or 50% of the daily needs with the midday meal [35].

## 4. Conclusions

In line with the uses of the past, the recovery of the school canteen and feeding model that was promoted from the Edalnu Programme may be of interest for current food and nutrition education policies.

The results shows how the school canteen became an educational tool, and the importance of considering gastronomic criteria in the promotion of healthy and sustainable eating habits. The aim was to use the table and the after-dinner meal as learning environments.

To achieve all these objectives, the school canteens, with the help of the food guide “the food wheel”, had planned and calculated menus that were nutritionally balanced, as well as being didactic, by explaining the reason for each food.

Dialogue was encouraged between cooks and experts in nutrition and dietetics, with the aim of proposing simple culinary techniques and guaranteeing harmony, balance, variety of ingredients, as well as the palatability of the dishes. The aim was to ensure their consumption and to educate the palate through a presentation that would make them pleasant and attractive.

The menus were eventually adapted to the gastronomic diversity of the Spanish regions, although without renouncing the objective of teaching how to eat new foods. Basically, the aim was to offer food that was close to the schoolchildren’s environment. At the same time as favouring the consumption of seasonal and local products, a culinary grammar such as that which characterises traditional Mediterranean food culture was encouraged, especially in the first courses.

Over the years, in addition to taking into account the problems of malnutrition due to excess that were beginning to emerge in the school environment, the recipe books were adapted to the changes in the way Spanish society ate and the complexity of the provision of school canteen services. Alternatives such as combined dishes were introduced. Although they covered the requirements in terms of nutrients, they broke with one of the strengths of traditional recipes: cereals ceased to be the base or main element, with pulses and vegetables as the main complements, and were replaced by meat or fish. This encouraged increased consumption of animal protein and fat, one of the risk factors that has contributed to the development of the current pandemic of overweight and obesity.

## Figures and Tables

**Table 1:** School lunches (first and second course, dessert, dairy complement or dried fruit and bread) elaborated in 1961.

1	Lentils with potatoes and carrots Fried whitebait with lettuce Dried figs, orange and bread	10	Soup. Chickpea stew with potatoes and chorizo Fried permit with lettuce Orange, milk and bread
2	Macaroni with tomato, chorizo and cheese Spanish omelette Orange, milk and bread	11	Potatoes with cod Minced-meat patties with lettuce Orange, milk and bread
3	Salad with lettuce, tomato and sardines Minced-meat patties with chips Orange, milk and bread	12	Beans with potatoes and carrots Fired eggs with béchamel sauce Orange, milk and bread
4	Stew (soup, chickpeas, carrot and chorizo) Meat from the stew in croquettes with chard Orange, milk and bread	13	White rice with peppers, tomatoes and chopped hard-boiled egg Fillet of meat with chard (or other combinations) Orange, milk and bread
5	Rice with tomato, peppers and meat Fried egg Orange, milk and bread	14	Potatoes, olives, carrot and mayonnaise Meatballs with chips Cheese, orange and bread
6	Red beans with chorizo Meat ragout with potatoes and carrot Orange, milk and bread	15	Rice with tomato, peppers and clams (or similar) Meatballs Orange, milk and bread
7	Macaroni with cheese and tomato Minced-meat (meatballs) and lettuce Orange, milk and bread	16	Noodle soup made with stock cubes Meat with potatoes and carrots Rice pudding and bread
8	Potato and lettuce salad Fried sardines Orange, milk and bread	17	White rice, tomato, peppers and fried egg Tuna with lettuce Orange, milk and bread
9	Lentils with chorizo and carrots Cod with Pil sauce Orange, milk and bread	18	For the summer Gazpacho Minced-meat patties with chips Orange, milk and bread

Source: *Plan de comedores escolares*. Madrid, Ministerio de Educación Nacional/Dirección General de Enseñanza Primaria. Servicio Escolar de Alimentación/Departamento de Comedores Escolares, 1961, capítulo IV (Dietética)-Anejo 1.

**Table 2:** Regional dishes for school canteens in 1966.

Region and Provinces	Recipes (First and Second Course), Complemented with Orange, Milk and Bread	Informer/Proposer Edalnu Diploma-Holders
Cantabria-Navarre: Asturias, Santander, Biscay, Guipúzcoa, Álava, Logroño and Navarre	Beans with potatoes and chopped carrots. Whiting (or similar in sauce).	Rafael Guerra Indurain (Navarra)
	Cream of broad beans and potatoes with chorizo and fried bread. Hard-boiled egg with tomato.	
	Lentils with rice and chorizo. Spanish omelette in sauce.	
	“Vigil” chickpeas. (Chickpea and vegetable stew). Bacon-coated whiting (or similar) and lettuce.	
Northern plateau: Soria, Segovia, Ávila, Salamanca, Valladolid, Palencia and Burgos	Ratatouille. Breaded liver fillets with chips.	Rosario Rojo Peraita (Burgos)
	Castilian cocido (stew).	
	Porrusalda (stew of potatoes, leeks and cod). Meatballs with tomato.	
Eastern coast: Balearic Islands, Alicante, Castellón, Valencia and Murcia	Fish and vegetable paella. Salad of lettuce, tomato and fish in oil.	Dolores Colvee Guillén (Valencia)
	Chicken and vegetable paella. Lettuce and tomato salad.	
	Rice with chard and beans. Fried egg and chips.	
	Rice in broth with chicken offal, clams and vegetables. Chicken in sauce with potatoes and carrot.	
Andalusia: Almería, Cádiz, Córdoba, Granada, Jaén, Huelva, Málaga and Seville entry 4	Gazpacho (bread and mayonnaise). Liver in onion sauce with chips.	José Pareja Mestres (Málaga)
	Noodle casserole with cod, tomato, peppers, potatoes and onion. Spanish omelette.	
	Lettuce salad. Andalusian stew.	
	“Migas” (fried breadcrumbs) with garlic, black pudding and chorizo. Fried sardines with lettuce.	
	Gazpacho. Minced-meat patties with chips.	
Canary Islands: Las Palmas and Tenerife	Rancho canario (legumes, potatoes, pasta, meat and cured meats). Tinned sardines with salad (tomato, onion and olives).	Antonio Hernández Gutiérrez (Tenerife)
	Bean stew. Salted fish with tomato and lettuce.	
Catalonia-Aragón: Barcelona, Gerona, Lérida, Tarragona, Huesca, Teruel, Zaragoza	Rice with cod, mussels, tomato, peppers and cheese. Fried egg and chips.	Rosario Vaquero Montemayor (Toledo)
	Noodle casserole. Tuna in oil with lettuce.	
	Catalan-style broad beans. Fried fish with lettuce.	
	Angler fish soup. Spanish omelette with lettuce.	
	Catalan-style rice. Omelette with tomato sauce.	
La Mancha and Extremadura: Albacete, Badajoz, Cáceres, Cuenca, Madrid and Toledo	Macaroni with tomato and cheese. Cod balls with lettuce.	Rosario Vaquero Montemayor (Toledo)
	Lentils with carrot, chorizo and lard. Extremaduran frité.	
	Migas manchegas (fried breadcrumbs). Hard-boiled egg with tomato.	
	Extremaduran gazpacho. Liver with lettuce.	
	Stewed potatoes with cod. Lamb stew.	

Source: Minutas regionales. In: *Plan de comedores escolares*. Madrid: Ministerio de Educación y Ciencia/Dirección General de Educación Primaria/Servicio Escolar de Alimentación y Nutrición, 1966, pp. 28–41.

**Table 3:** Meals (traditional dishes and combined dishes) for school canteens elaborated in 1985.

Traditional (Two Courses, Fruit and Dairy Complement)			
1	Stewed potatoes with meat Fillets of hake with salad Orange Milk	9	Potato casserole with white beans Roasted meat with salad Banana Milk
2	Cream of vegetables Hamburgers with sliced tomato Banana Milk	10	Green beans with tomato sauce Fried egg and chips Fruit salad Cheese
3	Spaghetti with tomato sauce and grated cheese Breaded liver with lettuce Orange Milk	11	Chickpea stew with carrots and spinach Hake in sauce Orange Milk
4	Picadillo soup (with vegetables, chicken, meat and egg) Roast beef with vegetables Orange Milk	12	Cauliflower with béchamel sauce Lamb chops with salad Orange Milk
5	Lentil stew Rioja-style omelette with salad Orange Milk	13	Chicken, ham and vegetable consommé Ham and chicken croquettes with tomato sauce Orange Milk
6	Valencian paella Cooked ham Orange Milk	14	Vegetable, chorizo and ham omelette Meat ragout Apple Cheese
7	Mixed vegetables with meat Battered hake with lettuce Banana Yoghurt	15	Boiled vegetables Pork loin with chips Orange Milk
8	Macaroni with chorizo and cheese Fried sole with salad Orange Milk		
Combined dishes			
1	Hard-boiled egg, tuna, potato, carrot, peas, green beans and ham. Vanilla ice-cream	4	Roast chicken, salad of lettuce, green beans, diced potatoes, carrots Orange juice Manchego cheese
2	Breaded fillet with chips, sliced tomato and lettuce Pear Custard	5	Rice mounds, fried egg and tomato sauce and lettuce Apple Cheese
3	Spanish omelette with tomato sauce, slices of cured sausage and a portion of cheese Orange	6	Minced-meat patties, fresh tomato and chips Orange juice Chocolate milkshake

Source: López Nomdedeu, Consuelo; Iglesias Reymunde, Teresa; Nájera Morrondo, Pilar. *Minutas para comedores escolares*. Madrid, Edalnu/Ministerio de Sanidad y Consumo/Dirección General de Salud Pública, 1985, pp. 12–39.

## Data Availability

Not applicable.
